# Evaluation of Maternal Ischemia-Modified Albumin Levels during Pregnancy and Their Effect on Fetal Birth Weight

**DOI:** 10.3390/medicina60091530

**Published:** 2024-09-19

**Authors:** Uğur Çobanoğlu, Özer Birge, Mustafa Çetin, Emine Seda Güvendağ Güven

**Affiliations:** 1 Department of Obstetrics and Gynecology, Private Ada Hospital, 28100 Giresun, Turkey; 2 Department of Gynecological Oncology, Niger Turkey Friendship Hospital, Niamey G3PR+2JJ, Niger; ozbirge@gmail.com; 3 Department of Obstetrics and Gynecology, Ordu Training and Educaiton Hospital, 52100 Ordu, Turkey; mustafacetin87@gmail.com; 4 Department of Obstetrics and Gynecology, Karadeniz Teknik University, 61100 Trabzon, Turkey; emine.seda@ktu.edu.tr

**Keywords:** ischemia-modified albumin, oxidative stress, fetal birth weight

## Abstract

*Background and Objectives*: The purpose of this study is to evaluate the impact of maternal ischemia-modified albumin (IMA) levels on pregnancy-related complications, fetal growth, and development over time. *Materials and Methods*: The prospective longitudinal and single-center study included 43 pregnant women ages 18 to 43. Routine pregnancy follow-up began at the first antenatal examination for all pregnant women before 14 weeks gestation, with IMA levels measured during the first, second, and third trimesters. The albumin cobalt binding test was used to determine the amount of ischemia-modified albumin (IMA). The patients’ medical, sociodemographic, and nutritional data were analyzed. The primary outcome was to investigate how changes in maternal ischemia affected albumin levels during pregnancy and the relationship between these changes and newborn weight. *Results*: This study included 43 cases with a mean age of 28.5 ± 5.2 years and a mean gestation period of 39.2 ± 1.3 weeks. The mean IMA levels for cases in the first trimester, second trimester, and third trimester were 0.53 ± 0.06, 0.64 ± 0.11, and 0.64 ± 0.06, respectively. The second and third trimesters showed significantly higher levels of ischemia-modified albumin (IMA) than the first trimester (*p* < 0.01). There was no statistically significant difference in IMA levels between the second and third trimesters (*p* = 1.000; *p* > 0.05). There was no statistically significant correlation between fetal birth and percentage changes in IMA measurements between the first and second trimesters, the first and third trimesters, or the second and third trimesters (*p* > 0.05). *Conclusions*: Our study determined that maternal ischemia-modified albumin levels during pregnancy did not correlate with fetal birth weight. Our findings revealed that age, sociodemographic changes, BMI, weight gain, and pregnancy complications had no effect on the change in IMA levels during pregnancy. We believe that this result will serve as a benchmark for future studies on IMA levels during pregnancy.

## 1. Introduction

During acute ischemia, albumin’s ability to bind transition metals such as copper, nickel, and cobalt decreases. Ischemia-modified albumin (IMA) is a modified form of albumin, created as a result of ROS (reactive oxygen species) action under reduced oxygen tension conditions in response to hypoxia or acidosis. During ischemia, the increased ROS generation inducing structural modification of albumin results in a reduction in its ability to bind endogenous and exogenous ions. Modification of its N-terminal amino acids (Asp-Ala-His) leads to decreased transition metal (Co^2+^, Ni^2+^, Cu^2+^) binding capacity of the albumin molecule, which is the basis of its measurement in biological fluids [1]. As a result, a metabolic albumin derivative known as ischemia-modified albumin (IMA) is produced. Ischemia-modified albumin level measurement has recently been proposed as an important diagnostic marker for myocardial ischemia. The U.S. Food and Drug Administration (FDA) has approved ischemia-modified albumin (IMA) as a serum biomarker for cardiac ischemia, which is used in the risk assessment of patients suspected of having acute coronary syndrome [2].

According to studies, pregnancy frequently occurs in a relatively hypoxic intrauterine environment, with subsequent reperfusion and oxidative stress playing an important role in trophoblast development [3,4]. Nonetheless, this scenario has not been conclusively demonstrated in a clinical setting using appropriate biochemical markers. Ongoing research is looking into the use of IMA as a biochemical marker to detect a hypoxic intrauterine environment.

IMA is used as a biomarker of ischemia in pathological clinical conditions like cardiac ischemia, pulmonary thromboembolism, lower extremity ischemia, and cerebrovascular events [5]. However, the physiological changes caused by this new biomarker during pregnancy have yet to be properly explained.

Increases in the IMA level detected in myocardial ischemia are also seen in pulmonary embolism, type II diabetes with poor glycemic control, acute blood loss, and following ischemia [6,7,8].

Studies have begun to investigate IMA level changes in the gynecological and obstetric fields, with a rise in serum IMA levels observed, particularly in preeclampsia patients experiencing placental hypoperfusion [9]. While some studies suggest that elevated maternal IMA levels may help assess neonatal hypoxia in cases of intra-uterine growth retardation (IUGR) [10], more research is needed to determine the role of IMA, a nonspecific biomarker, in gynecology and obstetrics.

Oxidative stress also plays a role in the pathophysiology and complications of gestational diabetes mellitus. Oxidative stress occurs with the formation of harmful free radicals in the body. Free radicals can be produced by many reactions required for the continuation of a normal metabolism and energy production in the cell. Toxic levels of free radicals interact with lipids, proteins, and nucleic acids, causing loss of membrane integrity, structural and functional changes in proteins, and genetic mutations. The body has some enzymatic and non-enzymatic antioxidant defense systems to cope with the effects of these harmful radicals. It is suggested that the low insulin sensitivity and high glucose levels seen in GDM may be the cause of oxidative stress and lead to the production of free radicals [11,12].

The purpose of this study is to analyze the change in maternal ischemia-modified albumin (IMA) levels during pregnancy and evaluate its relationship with pregnancy prognosis, pregnancy-related complications, and fetal growth and development.

## 2. Materials and Methods

Our prospective, single-center clinical analysis included 43 pregnant women aged 18 to 43 who sought antenatal pregnancy follow-up at Rize University Training and Research Hospital’s Pregnancy Polyclinic, part of the Department of Gynecology and Obstetrics. The participants signed the patient information and consent form, and the Local Ethics Committee approved this study (date: 10 March 2013, number: 2013/53).

Individuals with a prior medical history of ischemic disease, heart disease, Type I–II diabetes mellitus, hypertension, hypothyroidism, multiple pregnancies, abnormal serum albumin levels (normal range: 3–5.5 mg/dL), recurrent pregnancy loss, fetal anomalies, or abnormal troponin I levels or who were on a special diet as a result of their disease (gluten- or casein-free diet, vegetarian diet, liver) were excluded. Participants were excluded from this study if their pregnancies ended before the 24th week, if they missed routine prenatal follow-up appointments, or if their child was born outside of our medical facility. This study ended with the participation of 43 patients who met the predetermined study criteria.

### 2.1. Working Method

Our study was designed as prospective, longitudinal, and single center. All pregnant women (less than 14 weeks) began routine pregnancy follow-up with their first antenatal examination, and IMA levels were measured in the first, second, and third trimesters.

The patient follow-up form was filled out with medical, reproductive, and sociodemographic information from in-person interviews. The patient’s follow-up form included anthropometric measurements such as age, gravidity, parity, number of abortions, body weight, height, and body mass index (BMI) (kg/m^2^). The participants were divided into three categories based on their maternal education level: those with 0–8 years of education were classified as having a low education, those with 9–12 years of education were considered to have a middle education, and those with 13 years or more of education were classified as having a high education. The socioeconomic level was classified as good or bad based on the patient’s education level, occupation, spouse’s occupation, home ownership status, and household possessions [13].

During the first trimester, pregnant women’s nutritional status was assessed with the standardized “Food Consumption Form” [14]. Participants were instructed to complete the survey by documenting everything they ate and drank over the course of three days. The daily energy and nutrient intakes of women were determined using a computerized nutrition information system (BeBİS) program tailored to our country. These results were then compared with the recommended levels for pregnant women as outlined in the Nutrition Guide for Turkey. A dietary intake of 67–133% energy and nutrients are considered optimal nutrition. In contrast, falling below 67% indicates malnutrition, and exceeding 133% indicates overnutrition [15]. This study included information about whether patients received folic acid, iron, or other multivitamin supplements during pregnancy follow-ups.

The patients’ obstetric and neonatal outcomes were evaluated using follow-up and birth records, as well as neonatal unit data. Preeclampsia, gestational diabetes, intrauterine growth restriction, and premature birth rates were calculated. The newborn’s week of birth, type of birth, birth weight, and Apgar 1 and 5 min assessments were recorded in the postnatal period.

### 2.2. Ischemia-Modified Albumin

Blood samples were collected from the antecubital vein between 8:00 and 10:00 a.m., following an overnight period of fasting. Blood samples were collected in gel tubes, allowed to clot, and then centrifuged at 3000 rpm for 10 min to separate serums for biochemistry testing. The albumin cobalt binding test was used to determine the amount of ischemia-modified albumin (IMA). The reduced affinity of cobalt for albumin was determined using the fast and colorimetric measurement method developed by Bar-Or et al. [13]. Patient serum (200 mL) was slowly mixed with 50 mL of 0.1% CoCl2.6H_2_O (Sigma) in glass tubes. The mixture was gently stirred and incubated for 10 min to allow for proper cobalt albumin binding. To color, 50 mL of a 1.5 mg/mL solution of dithiothreitol (DTT) (Sigma) was added. After 2 min, the process was interrupted by adding 1 mL of 0.9% NaCl to prevent cobalt and albumin binding. A blank sample was made for each individual sample. A serum cobalt blank without DTT was created by replacing 50 mL of 1.5 mg/mL dithiothreitol (DTT) with 50 mL of distilled water at the point of DTT introduction. The absorbance of the samples was measured at 470 nm using a Shimadzu UV1601 spectrophotometer. Color formation in the DTT samples was compared with that in the blank tubes, and the results were expressed as ABSU absorbance units.

### 2.3. Statistical Evaluation

The statistical analysis was carried out using NCSS (Number Cruncher Statistical System) 2007 and PASS (Power Analysis and Sample Size) 2008 Statistical Software programs from Kaysville, UT, USA. The study data were analyzed using descriptive statistical methods such as mean, standard deviation, frequency, and ratio. Follow-up measurements of normally distributed quantitative data parameters were also performed using analysis of variance in repeated measurements, paired sample *t*-tests, or pairwise comparisons. The Mann–Whitney U test was used to compare non-normally distributed parameters between two groups, while the Kruskal–Wallis test was used to compare three groups. In addition, the Mann–Whitney U test was used to identify the group responsible for the observed discrepancy. Spearman’s correlation analysis was used to determine the correlations between parameters. Significance was determined at the *p* < 0.05 and *p* < 0.01 levels.

## 3. Results

This study examined 43 cases with a mean age of 28.5 ± 5.2 years and a gestation duration of 39.2 ± 1.3 weeks. This study found that participants had a mean of 2.56 ± 1.37 pregnancies, 1.14 ± 1.18 parity, and 0.42 ± 0.66 abortions. Of all the cases, 53.5% (*n* = 23) had a maternal education level of 13 or more years; 14% (*n* = 6) had an education level of 9–12 years; and 32.6% (*n* = 14) had an education level of 0–9 years. The participants’ mean pre-pregnancy weight was 65.1 ± 11.1 kg (48–100), and their mean birth weight was 81.3 ± 12.4 kg (62–115). The study participants’ fetal birth weights ranged from 2800 to 4600 grammes, with a mean of 3391.40 ± 359.34. Fourteen percent (*n* = 6) of the patients in this study experienced pregnancy-related complications. Among the participants, 7% (*n* = 3) had intrauterine growth restriction, 7% (*n* = 3) had preterm delivery, 4.7% (*n* = 2) had preeclampsia, and 14% (*n* = 6) had gestational diabetes. Of the cases included in this study, 97.7% (*n* = 42) had adequate nutritional status during pregnancy, while 2.3% (*n* = 1) had under-nutrition nutritional status. Among the participants, 55.8% (*n* = 24) had normal births, while 44.2% (*n* = 19) had caesarean sections (Table 1).

The mean IMA measurements of the study participants were 0.53 ± 0.06 (0.408–0.656) in the first trimester, 0.64 ± 0.11 (0.519–1.289) in the second trimester, and 0.64 ± 0.06 (0.466–0.769) in the third trimester. The study participants’ first trimester maternal weights varied from 49 to 102 with a mean of 66.58 ± 10.77 and 110 with a mean of 76.21 ± 11.62. The Apgar score for all newborn babies appears to be within normal limits, with the 1st minute mean being 7 and the 5th minute mean being 9 (Table 2).

A statistically significant difference was identified in the IMA measurements of the study participants based on trimesters (*p* < 0.01). Based on the first trimester IMA measurements, it was determined that the mean IMA change of −0.107 ± 0.12 units in the second trimester and −0.105 ± 0.07 units in the third trimester were found to be highly statistically significant (*p* = 0.001, *p* = 0.001; *p* < 0.01). No statistically significant change was detected between the IMA levels of the second trimester and the third trimester (*p* = 1.000; *p* > 0.05). A statistically significant difference was identified in the maternal weight measurements of the study participants based on trimesters (*p* < 0.01). The mean maternal weight change of 4.58 ± 0.40 units in the second trimester and 9.63 ± 0.85 units in the third trimester was significantly statistically different from the first trimester (*p* = 0.001, *p* = 0.001; *p* < 0.01). It was determined that the mean change of 5.04 ± 0.55 units in maternal weight between the second and third trimesters was found to be highly statistically significant (*p* = 0.001). The results of the 75 g OGTT for each case are shown in Table 3.

A weak correlation was found between age and the percentage change in IMA measurements between the first and second trimesters; however, this was not statistically significant (r = 0.129; *p* > 0.05). There was no statistically significant relationship between age and the percentage change in IMA measurements in the first and third trimesters, or between the second and third trimesters (*p* > 0.05).

**Figure 1:**
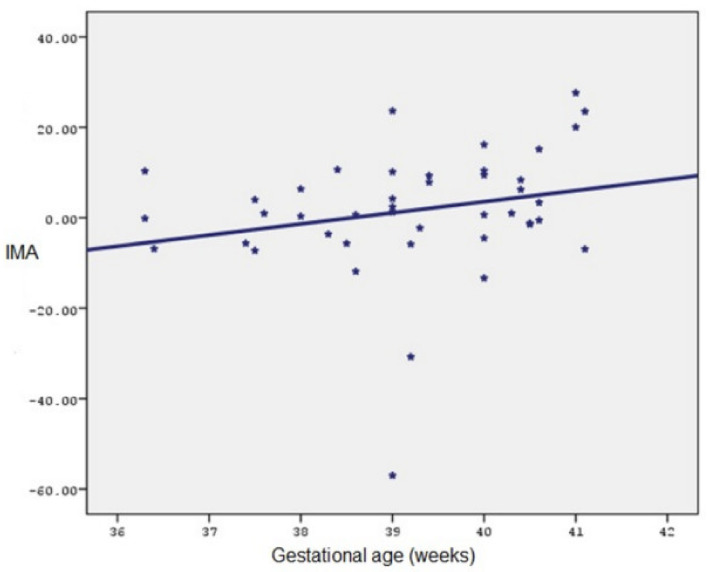
Relationship between gestational age and II–III trimester IMA percentage change (Pearson correlation coefficient 0.306).

A weak negative relationship was found between gestational age and percentage change in IMA measurements in the first and second trimesters, but it was not statistically significant (r = −0.148; *p* > 0.05). A weak positive relationship was found between gestational age and percentage change in IMA measurements in the first and third trimesters, but it was not statistically significant (r = 0.176; *p* > 0.05). A positive, statistically significant relationship was found between gestational age and the percentage change in IMA measurements in the second and third trimesters (r = 0.306; *p* < 0.05) (Figure 1).

There was no statistically significant relationship between pre-pregnancy weight and percentage change in IMA measurements during the first and second trimesters (*p* > 0.05). There was no statistically significant relationship between pre-pregnancy weight and percentage change in IMA measurements in the first and third trimesters, or between second and third trimesters (*p* > 0.05).

There was no significant relationship between maternal weight at birth and percentage change in IMA measurements in the first and second trimesters, the first and third trimesters, and the second and third trimesters (*p* > 0.05).

There was no statistically significant relationship between fetal birth weight and percentage of IMA measurements in the first and second trimesters, the first and third trimesters, and the second and third trimesters (*p* > 0.05) (Table 4).

According to gravida numbers, there was no statistically significant difference in the percentage change in IMA measurements between the first and second trimesters, the first and third trimesters, and the second and third trimesters (*p* > 0.05).

There was no statistically significant difference in the percentage change in IMA measurements between the first and second trimesters, the first and third trimesters, or the second and third trimesters based on smoking status during pregnancy (*p* > 0.05).

There were no statistically significant differences in the percentage change in IMA measurements between the first and second trimesters, the first and third trimesters, or the second and third trimesters, according to the patients’ socioeconomic status (*p* > 0.05).

In 41 cases who used vitamins during pregnancy, the mean II–I trimester IMA percentage change was 21.19 ± 24.18, the III–I trimester IMA percentage change was 21.26 ± 16.28, and the III–II trimester IMA percentage change was 1.95 ± 14.27. A comparison could not be made because vitamins were not used in two cases.

In 42 cases with good nutritional status during pregnancy, the mean II–I trimester IMA percentage change was 21.62 ± 23.76, the mean III–I trimester IMA percentage change was 21.21 ± 16.15, and the mean III–II trimester IMA percentage change was 1.49 ± 14.24. A comparison could not be made because one of the cases had low nutritional status during pregnancy.

There was no statistically significant difference in the percentage change in IMA measurements between the first and second trimesters, the first and third trimesters, or the second and third trimesters based on maternal education level (*p* > 0.05) (Table 5).

There was no statistically significant difference in the percentage change in IMA measurements between the first and second trimesters, the first and third trimesters, or the second and third trimesters in terms of pregnancy complications (*p* > 0.05).

In two cases of intrauterine growth restriction, the mean II–I trimester IMA percentage change was 10.54 ± 18.08, while the mean III–I trimester IMA percentage change was 22.50 ± 10.12, and the III–II trimester IMA percentage change was 12.27 ± 14.60. There were two cases of intrauterine growth restriction, so a comparison was not possible.

In three preterm birth cases, the mean II–I trimester IMA percentage change was 23.71 ± 26.78, the mean III–I trimester IMA percentage change was 24.38 ± 24.52, and the III–II trimester IMA percentage change was 1.09 ± 8.66. A comparison could not be made because there were only three cases of preterm birth.

In two cases of preeclampsia, the mean II–I trimester IMA percentage change was 36.95 ± 22.60, the mean III–I trimester IMA percentage change was 35.96 ± 23.67, and the mean III–II trimester IMA percentage change was −0.80 ± 0.91. A comparison could not be made because there were two cases of preeclampsia present.

According to the presence of gestational diabetes, there was no statistically significant difference in percentage change in IMA measurements between the first and second trimesters, the first and third trimesters, and the second and third trimesters (*p* > 0.05) (Table 6).

## 4. Discussion

Our study aims to evaluate the levels of ischemia-modified albumin, a new ischemic marker, in the first, second, and third trimesters of pregnancy and determine its relationship to fetal birth weight.

Our study found a weak association between age and the percentage change in IMA measurements during the first, second, and third trimesters, but it was not statistically significant. According to gravida numbers, there was no statistically significant difference in percentage change in IMA measurements between the first and second trimesters, the first and third trimesters, and the second and third trimesters. There was no statistically significant difference in percentage change in IMA measurements between the first and second trimesters, between the first and third trimesters, and between the second and third trimesters based on fetal birthweight.

Ischemia-modified albumin is a new marker used in the diagnosis of coronary artery disease in recent years. In the late 1990s, researchers discovered that patients with acute coronary syndrome had lower levels of exogenous cobalt (Co^+2^) binding to human serum albumin. This metabolic variant, known as ischemia-modified albumin, was measured using the “Albumin Cobalt Binding” test [16,17]. The IMA has also received FDA approval for this purpose. IMA is detected in serum within 10 min of ischemia. This is a relatively short period of time given the presence of myoglobulin, CK-MB, and troponin-C in the blood. Some studies have shown that IMA, along with troponin and ECG, has a 95% accuracy for diagnosing acute coronary events [18]. None of the pregnant women included in our study had a history of cardiac ischemic disease.

Today, IMA is recognized as a useful marker in a variety of situations, including myocardial ischemia, skeletal muscle ischemia, mesenteric ischemia, stroke, and cerebrovascular ischemia [19]. It is used to diagnose acute ischemic diseases in men and non-pregnant women. There is insufficient information in the literature about the physiological changes in IMA levels during pregnancy and the impact of these changes on pregnancy prognosis. Güven et al. found that levels of IMA increase during pregnancy [20]. This can be explained by the fact that physiological oxidative stress increases during pregnancy [21].

The identification of IMA levels during pregnancy was the most important finding of our pilot study. IMA levels were significantly lower in the first trimester when compared with the second and third trimesters. Güven et al. conducted a study and found similar results in IMA levels tested in maternal serum across various patient groups during the first, second, and third trimesters of pregnancy [20]. The rise in IMA levels during the second and third trimesters of pregnancy can be attributed to the fetoplacental unit’s increased need for substrates and metabolism, as well as changes in the oxidant/antioxidant balance. 

IMA levels rise during pregnancy compared with the period before pregnancy [20]. This increase in the first trimester of pregnancy is most likely caused by placentation. A hypoxic environment within the uterus is created by the invasion of extra villous trophoblasts. This is a physiological reaction required for the normal progression of pregnancy. During pregnancy, oxidative stress levels increase [21].

Prefumo et al. found that levels of IMA increased during the first trimester of pregnancy compared with the period before pregnancy [22]. The physiological transformation of the maternal spiral arteries requires extra villous trophoblast invasion, according to the researchers. The hypoxic intrauterine environment caused by plugs forming in the spiral arteries may be the result of this process.

Our study found that in the two cases of preeclampsia, the mean percentage change in IMA levels was 36.95 ± 22.60 in the II–I trimester, 35.96 ± 23.67 in the III–I trimester, and −0.80 ± 0.91 in the III–II trimester. A comparison could not be conducted because there were two cases of preeclampsia. In the first trimester of pregnancy, Papageorghiou et al. found that levels of IMA were significantly higher in those who later developed preeclampsia than in those with a normal pregnancy prognosis [23]. This finding supports the hypothesis that increased uterine oxygen deprivation in the uterus and the resulting oxidative stress during reperfusion impair trophoblast growth and contribute to the development of preeclampsia. The first-trimester IMA findings presented in this study will be useful for future research into abnormal placental development. Gafsou et al. discovered significantly higher IMA findings in cases of preeclampsia than in patients with a normal pregnancy prognosis [24]. Lacovidou et al. discovered that fetal cord IMA levels in pregnant women with IUGR were similar to cord blood levels in pregnant women with normal pregnancy prognosis [25].

Kumral et al. found that fetal cord blood IMA levels were significantly higher in those with pregnancy complications compared with those without [26]. They also stated that IMA levels were significantly higher in individuals with Doppler results indicating perinatal asphyxia. 

Our study found no statistically significant effect of IMA changes or IMA levels on infant birth weight. Furthermore, no significant correlation was found between IMA levels and infant birth weight in any of the pregnancy trimesters. Golbaşı et al. (2022) compared IUGR fetuses and 227 infants with normal weight but found no significant relationship between infant birth weight and maternal IMA samples obtained at birth [27].

Jaiswar et al. (2022) examined IMA levels, infant birth weight, and placental histopathology in 80 pregnant women, 40 of whom were normotensive and 40 of whom had preeclampsia. This study’s findings revealed a significant relationship between maternal IMA levels and placental histopathology. Furthermore, a significant relationship was discovered between maternal IMA levels and infant birth weight when comparing fetuses weighing less than 2 kg with those weighing more than 2 kg at birth. When comparing fetuses weighing more than 2 kg with those weighing more than 2.6 kg, the results revealed no significant relationship between infant birth weight and maternal IMA levels [9].

Güven et al. examined IMA levels in 117 pregnant and 23 non-pregnant healthy women across three trimesters of pregnancy. Pregnant women’s blood levels, fetal birth weights, malondialdehyde levels, and albumin levels were all analyzed over three trimesters. Researchers discovered that pregnant women had higher levels of IMA in their blood than healthy individuals. Accordingly, they concluded that pregnancy is a process with physiological oxidative stress compared with normal conditions. They also concluded that normal limits will be different during pregnancy when IMA is used as a marker in acute ischemic situations during pregnancy. An increase in maternal IMA levels was seen in the second and third trimesters compared with the first trimester [20]. Our findings are consistent with these results.

Upon reviewing our study, we discovered that age, sociodemographic changes, BMI, weight gain, and pregnancy complications had no influence on the change in IMA levels during pregnancy. We believe that this result will serve as a benchmark for future studies on IMA levels during pregnancy.

Our study has some limitations. The limited number of patients in this study precludes drawing significant conclusions about the potential associations between IMA findings and pregnancy complications. In addition, although we evaluated the IMA changes in pregnant women according to the trimesters in our study, the absolute effect of pregnancy could not be clearly revealed as a result of not comparing the absolute IMA values of healthy women before pregnancy and women in the post-pregnancy period, which constitutes a limitation of our study.

This paper’s strengths include a longitudinal design, relatively comprehensive obstetric and demographic phenotyping of participants, and the reporting of significant adverse data.

## 5. Conclusions

The most important result of this pilot study was the determination of IMA levels during pregnancy. Compared with the second and third trimesters, IMA levels were significantly reduced in the first trimester. A substantial number of randomized controlled studies are required to determine whether the rise in IMA levels during the second and third trimesters of pregnancy is attributable to an increase in fetoplacental unit substrate demand and metabolism, as well as a shift in the oxidant/antioxidant balance.

In our study, we observed that the change in IMA levels during pregnancy was not influenced by factors such as age, sociodemographic changes, BMI, weight gain, and pregnancy complications. We believe that this result will serve as a benchmark for future research on IMA levels in pregnancy.

There is no relationship between the percentage change in IMA measurements and the fetal birth weight during the first and second trimesters, the first and third trimesters, and the second and third trimesters, according to another finding of our study.

## Figures and Tables

**Table 1:** Demographic and clinical characteristics of the cases.

		(Median) Min–Max/N	Mean ± SD/%
Age (year)		31 (20–41)	
Body height (cm)		162 (151–172)	
Gravida		4 (1–7)	
Parity		3 (0–5)	
Abortus		2 (0–3)	
Live birth		3 (0–5)	1.09 ± 1.17
Duration of pregnancy (week)		37 (36–41)	39.21 ± 1.32
Pre-pregnancy weight (kilogram)		73 (48–100)	65.16 ± 11.19
Maternal weight at birth (kilogram)		85 (62–115)	81.35 ± 12.48
Fetal birth weight (gram)		3450 (2800–4600)	3391.40 ± 359.34
Complication in pregnancy		6 (14%)	
Intrauterine growth restriction (IUGR)		3 (7%)	
Preterm birth		3 (7%)	
Preeclampsia		2 (4.7%)	
Gestational diabetes mellitus (GDM)		6 (14%)	
Smoking in pregnancy		6 (14%)	
Vitamin supplementation in pregnancy		41 (95.3%)	
Nutritional level in pregnancy		42 (97.7%)	
Type of birth	Vaginal	24 (55.8%)	
	Cesarean	19 (44.2%)	

**Table 2:** Maternal serum levels of ischemia-modified albumin of trimesters.

	Min–Max	Mean ± SD
I. Trimester IMA results	0.408–0.656	0.53 ± 0.06
II. Trimester IMA results	0.519–1.289	0.64 ± 0.11
III. Trimester IMA results	0.466–0.769	0.64 ± 0.06
I. Trimester maternal weight	49–102	66.58 ± 10.77
II. Trimester maternal weight	52–105	71.16 ± 10.94
III. Trimester maternal weight	56–110	76.21 ± 11.62
75 gr OGTT ˣ at 0 Min.	77–100	86.05 ± 6.10
75 gr OGTT at 60 Min.	82–261	131.67 ± 35.38
75 gr OGTT at 120 Min.	49–178	111.35 ± 25.28

ˣ Oral Glucose Tolerance Test (OGTT).

**Table 3:** Evaluation of IMA, maternal weight, and OGTT test results according to trimesters.

	IMA		
	Mean	SD	^a/b^ *p*
I. Trimester	0.53	0.06	0.001 **
II. Trimester	0.64	0.11	
III. Trimester	0.64	0.06	
	Maternal Weight		
I. Trimester	66.58	10.77	
II. Trimester	71.16	10.94	0.001 **
III. Trimester	76.21	11.62	
	OGTT		
OGTT at 0 Min.	86.05	6.09	
OGTT at 60 Min.	131.67	35.37	0.001 **
OGTT at 120 Min.	111.35	25.28	

^a^ Repeated measure test. ^b^ Adjustment for multiple comparisons: Bonferroni test. ** *p* < 0.01.

**Table 4:** Age, gestational age, pre-pregnancy weight, maternal weight at birth and fetal birth weight, and IMA percentage changes in trimesters.

	Age-IMA % Changes
	R
I.—II. Trimester IMA % changes	0.129
I.—III. Trimester IMA % changes	−0.074
II.—III. Trimester IMA % changes	−0.173
	Gestational age-IMA % changes
I.—II. Trimester IMA % changes	−0.148
I.—III. Trimester IMA % changes	0.176
II.—III. Trimester IMA % changes	0.306
	Pre-pregnancy weight-IMA % Changes
I.—II. Trimester IMA % changes	0.062
I.—III. Trimester IMA % changes	0.102
II.—III. Trimester IMA % changes	−0.070
	Maternal weight at birth-IMA % changes
I.—II. Trimester IMA % changes	0.014
I.—III. Trimester IMA % changes	−0.015
II.—III. Trimester IMA % changes	−0.121
	Fetal birth weight-IMA % changes
I.—II. Trimester IMA % changes	−0.091
I.—III. Trimester IMA % changes	0.021
II.—III. Trimester IMA % changes	0.150

R = Spearman correlation coefficient.

**Table 5:** Evaluation of IMA percentage changes in trimesters according to descriptive characteristics.

		I. Tri.—II. Tri. IMA % Changes	I. Tri.—III. Tri. IMA % Changes	II. Tri.—III. Tri. IMA % Changes
		Mean ± SD (Med.)	Mean ± SD (Med.)	Mean ± SD (Med.)
Gravida	1 (*n* = 10)	27.65 ± 42.51 (17.37)	18.65 ± 16.08 (10.36)	−1.36 ± 20.61 (4.32)
	>2 (*n* = 33)	19.31 ± 14.36 (17.69)	21.72 ± 16.18 (21.19)	2.51 ± 11.72 (0.96)
	^c^ *p*	0.908	0.314	0.818
Smoking in pregnancy	Yok (*n* = 37)	21.57 ± 24.58 (17.86)	20.27 ± 15.75 (19.22)	0.98 ± 14.84 (0.96)
	Var (*n* = 6)	19.29 ± 18.01 (16.15)	25.55 ± 18.42 (25.99)	5.47 ± 7.80 (5.92)
	^c^ *p*	0.674	0.649	0.344
Socioeconomic status	Good (*n* = 35)	22.32 ± 25.29 (20.97)	21.93 ± 16.10 (21.19)	1.88 ± 15.28 (2.41)
	Poor (*n* = 8)	16.57 ± 14.34 (15.72)	16.94 ± 16.01 (12.24)	0.39 ± 7.39 (−0.10)
	^c^ *p*	0.349	0.261	0.303
Vitamin supplementation in pregnancy	Yok (*n* = 2)	22.49 ± 0.54 (22.49)	15.80 ± 10.63 (15.80)	−5.44 ± 9.10 (−5.44)
	Var (*n* = 41)	21.19 ± 24.18 (16.88)	21.26 ± 16.28 (19.22)	1.95 ± 14.27 (1.25)
	*p*	-	-	-
Nutrition level in pregnancy	Good (*n* = 42)	21.62 ± 23.76 (17.78)	21.21 ± 16.15 (19.46)	1.49 ± 14.24 (0.98)
	Poor (*n* = 1)	5.51	12.22	6.36
	*p*	-	-	-
Maternal education level	≥13 Years (High) (*n* = 23)	23.81 ± 28.39 (17.86)	20.00 ± 18.14 (18.12)	−0.59 ± 16.49 (2.41)
	9–12 Years (Medium) (*n* = 6)	12.98 ± 10.27 (13.86)	16.42 ± 9.14 (14.99)	3.62 ± 11.18 (−1.33)
	0–9 Years (Low) (*n* = 14)	20.58 ± 18.75 (19.40)	24.62 ± 14.64 (22.03)	4.36 ± 10.67 (0.83)
	^d^ *p*	0.523	0.408	0.957

**^c^** Mann–Whitney U Test. ^d^ Kruskal–Wallis Test.

**Table 6:** Evaluation of IMA percentage changes in trimesters according to pregnancy complications.

		II. Tri.—I. Tri. IMA % Changes	III. Tri.—I. Tri. IMA % Changes	III. Tri.—II. Tri. IMA % Changes
		Mean ± SD (Med.)	Mean ± SD (Med.)	Mean ± SD (Med.)
Complication in pregnancy	No (*n* = 37)	20.57 ± 24.18 (16.69)	20.84 ± 16.18 (18.12)	2.16 ± 14.72 (2.41)
	Yes (*n* = 6)	25.45 ± 21.11 (24.14)	22.04 ± 16.37 (20.21)	−1.77 ± 9.56 (−2.53)
	^c^ *p*	0.262	1.000	0.139
Intrauterine growth restriction (IUGR)	No (*n* = 40)	22.05 ± 23.95 (17.29)	20.89 ± 16.46 (18.91)	0.81 ± 13.91 (0.98)
	Yes (*n* = 3)	10.54 ± 18.08 (20.97)	22.50 ± 10.12 (19.22)	12.27 ± 14.60 (10.63)
	*p*	-	-	-
Preterm birth	No (*n* = 40)	21.06 ± 23.72 (17.29)	20.75 ± 15.64 (19.46)	1.65 ± 14.49 (1.12)
	Yes (*n* = 3)	23.71 ± 26.78 (17.86)	24.38 ± 24.52 (10.69)	1.09 ± 8.66 (−0.16)
	*p*	-	-	-
Preeclampsia	No (*n* = 41)	20.48 ± 23.65 (16.88)	20.27 ± 15.61 (18.12)	1.72 ± 14.42 (1.25)
	Yes (*n* = 2)	36.95 ± 22.60 (36.95)	35.96 ± 23.67 (35.96)	−0.80 ± 0.91 (−0.80)
	*p*	-	-	-
Gestational diabetes mellitus (GDM)	No (*n* = 37)	22.20 ± 24.40 (17.69)	22.29 ± 16.73 (19.69)	1.96 ± 14.63 (1.25)
	Yes (*n* = 6)	15.35 ± 18.56 (19.30)	13.06 ± 7.15 (12.24)	−0.56 ± 10.92 (−4.06)
	^c^ *p*	0.700	0.132	0.309

**^c^** Mann Whitney U Test.

## Data Availability

The datasets used and/or analyzed during the present study are available from the corresponding author upon reasonable request.
